# Assessing Variations in Positional Performance across Age Groups and during Matches in Youth Association Football Competitions

**DOI:** 10.3390/s24144536

**Published:** 2024-07-13

**Authors:** Quanchen Liu, Zhuhang Huang, Diogo Coutinho, Xiaobin Wei, Tao An, Bruno Gonçalves

**Affiliations:** 1 China Football College, Beijing Sport University, Beijing 100084, China; quanchen1993@outlook.com; 2 Department of Sports Sciences and Physical Education, University of Maia, 4475-690 Maia, Portugal; dcoutinho@umaia.pt; 3 Research Center in Sports Sciences, Health Sciences and Human Development, CIDESD, 5000-801 Vila Real, Portugal; 4 School of Strength and Conditioning Training, Beijing Sport University, Beijing 100084, China; weixiaobin@bsu.edu.cn; 5 Sport Development Center of Tengzhou City, Tengzhou 277500, China; tztyzx@163.com; 6 Departamento de Desporto e Saúde, Escola de Saúde e Desenvolvimento Humano, Universidade de Évora, 7004-516 Évora, Portugal; bgoncalves@uevora.pt; 7 Comprehensive Health Research Centre (CHRC), Universidade de Évora, 7004-516 Évora, Portugal; 8 Portugal Football School, Portuguese Football Federation, 1495-433 Oeiras, Portugal

**Keywords:** global position system, collective behaviour, tactical analysis, youth players

## Abstract

This study aimed to explore how positional performance varies across different youth age groups and during matches in football competitions. The study encompassed 160 male outfield youth football players (*n* = 80, under-13, U13; *n* = 80, under-15, U15) who belonged to the starting line-up and played the entire first half of each match. The players’ positional data were gathered through the global positional system for each of the eight matches performed by each age group. The frequency of near-in-phase synchronization based on speed displacements, spatial exploration index, and the distance to the nearest teammate and opponent were used as variables. Additionally, each match half was segmented into three equal parts to assess changes over time and used as a period factor along with age group. The results indicated that U13 players showed a significant decrease (from small to large ES) in synchronization speed and spatial exploration index throughout the first half of the match, along with a decrease in the distance to the nearest opponent. In contrast, U15 players exhibited most changes during the third segment of the half, with a decrease in speed synchronization and spatial exploration, but an increase in the distance and regularity to the nearest opponent. Comparing both age groups revealed significant differences in speed synchronization across the entire half of the match and within each segmented period (from small to large ES), with U13 consistently showing higher values. The study highlights that long durations in 11 vs. 11 matches might not provide an appropriate learning environment in the U13 age group. Conversely, the U15 group displayed better capacity for tactical adjustments over time, suggesting a higher level of tactical maturity. Overall, these findings emphasize the importance of adapting youth football training and competition structures to the developmental needs and capabilities of different age groups to optimize learning and performance outcomes.

## 1. Introduction

One major challenge in sports science is identifying performance determinants to enhance coaching and competition outcomes [1]. Performance analysis plays a crucial role here, focusing on gathering valid, accurate, and reliable data during competitions to boost individual or team performance [2]. As one of the traditional methods of performance analysis in team sports, notational analysis seeks to obtain indicators of discrete actions and/or events by using advanced statistical procedures [3,4,5]. However, this method often fails to provide information regarding the dynamic confrontation of forces between the players and teams [6]. That is, teams continuously adjust and adapt their movement behaviour as a result of the cooperative (i.e., teammates’ actions) and competitive interactions (i.e., opposition movements). This means that a team may dictate the game rhythm during the earlier phase of the match; however, as the match unfolds, it is likely that the opposing team will adjust their positioning and actions to balance the match [7]. In general, the discrete performance indicators captured by traditional notational analysis would fail to capture these coordinative tendencies between both teams [8]. As a result of recent technological developments, research in sports sciences started to analyse players’ positioning dynamics, which considers the spatiotemporal relationships between both teams as a result of the collective principles of play, the opponents’ behaviour, and the contextual circumstances [8,9,10]. Consequently, analysing players’ positioning dynamics across the match seems to provide a more functional, holistic, and complex understanding of teams’ sports performance.

In association football, performance analysis should be a comprehensive process involving precise measurements of physiological, technical, and tactical workloads that ultimately influence player and team outcomes [6]. The physical and physiological demands of the players when involved in real practice scenarios have been investigated incessantly over the last years, describing the movement patterns during training [11,12,13] and competition environments [14,15,16]. Nevertheless, these demands seem to be very sensitive to the teams’ strategies, contextual variables, and opponent behaviour, indicating that multiple factors could impact players’ physical responses during matches [1,17,18]. For example, lower external load has been reported in teams that show higher positioning synchronization during training sessions [19], shedding light on the role of positioning and tactical behaviour on the players’ physical load. Positioning synchronization consists of a metric that measures the percentage of time that each pair of players moves in the same direction (e.g., the defensive line moving forward to follow the midfielders’ and strikers’ pressure) [9,19]. This variable has been used to distinguish teams’ quality, as the winning team seems to possess higher values of movement synchronization [20]. More recently, rather than players’ positioning, synchronization has been applied to players’ movement speed. In this context, Gonçalves et al. [10] showed that higher dyadic synchronization at high speeds in the first half periods may limit players’ performance in the second half. Accordingly, it was found a decrease in speed synchronization during the second half periods that may result from accumulated muscular and mental fatigue towards the match. Additionally, an examination of teams’ behaviour across 15 min intervals during a single match revealed variations in team dispersion throughout these periods, with more regular patterns emerging toward the match’s conclusion [7]. Altogether, the results from the previous study suggest that the integration of players’ physical performance with the collective principles of play may be achieved by analysing the synchronization speed. Additionally, exploring the players’ and team’s performance across time periods for each half (e.g., blocks of 15 min) would contribute to a better understanding of their performance.

In fact, the analysis of positional dynamics aims to identify and describe emergent tactical patterns that underpin performance, while preserving the sequential and situational characteristics of match events [8,9,21,22]. This means that while analysing players’ movements, researchers and data analysts must be aware that tactical patterns are dynamic and shift throughout the match, influenced by players’ varying capacities and external factors such as pre-match coaching strategies that guide collective behaviour [23]. However, as the match unfolds, players and teams are likely to adapt to changing play configurations and opposition strategies, which are known as tactics [24]. Thus, analysing players’ tactical performance during shorter periods can provide additional insights into how tactical decisions are executed under varying levels of fatigue [25,26].

Most research developed with positioning data has been applied to elite and adult levels. While studies examining youth players’ tactical behaviour exist, they are predominantly centred around training sessions [27]. For example, Olthof, Frencken [28] compared under-13 (U13), under-15 (U15), under-17 (U17), and under-19 (U19) performance during small-sided games (SSGs) while varying the pitch size. The authors found that an increase in the pitch size contributed to a higher external load, and also bigger distances between teams [28]. In addition, higher variability was found in players’ distances in larger formats [28]. This finding is especially important, as there has been a focus of discussion resulting from which size and playing format may be more appropriate for youth football players [29]. In fact, it is still common to find younger age groups (e.g., U13 and U15) playing 11-a-side in regular formats (e.g., length x width, 106 × 65 m playing area), which may not be appropriate for their development stage [29]. Despite the competitive setting concerns in youth football, research exploring their positional performance during matches is scarce. In fact, the limited available research exploring competitive formats in youth football has mostly compared it with SSGs [30]. Thus, exploring youth players’ positioning performance across different time periods in competitive settings while comparing different age groups may help responsible bodies and entities to better frame competition for youth players. In addition, larger playing spaces seem to induce large variability in their behaviour [28]. It may also be expected to see a higher variability when playing during long periods (e.g., one half), while also resulting in lower tactical knowledge when compared to older levels [31]. Thus, this study aimed to explore how positional performance varies across different youth age groups (i.e., U13 and U15) and time periods during competitive matches.

## 2. Materials and Methods

### 2.1. Participants

The study encompassed 160 male outfield youth football players, with 80 participants U13 belonging to eight teams (U13: average age 12.5 ± 0.5 years; average height 163.2 ± 8.2 cm; average weight 48.9 ± 6.7 kg; average playing experience 4.3 ± 1.7 years) and 80 from U15, also belonging to eight teams (U15: average age 14.5 ± 0.5 years; average height 169.1 ± 9.5 cm; average weight 53.7 ± 7.1 kg; average playing experience 6.5 ± 1.4 years). The U13 teams engaged in three weekly training sessions (approximately 90 min each) and played an official 11-a-side game on weekends. Similarly, the U15 teams participated in four weekly training sessions (around 90 min each) and competed in an official 11-a-side game on weekends. Goalkeepers were involved in the study but excluded from data analysis due to their specialized positional constraints and unique game dynamics compared to outfield players. Informed consent was obtained from coaches, players, parents, and the club prior to the study’s commencement. All participants were informed of their right to withdraw from the study at any time. The study’s procedures were approved by the local Institutional Research Ethics Committee and conformed to the Declaration of Helsinki guidelines.

### 2.2. Procedures and Instruments

The teams involved in the study participated in eight official matches as part of the Second China Youth Football League 2023, with each age group (U13 and U15) playing four matches. The analysis focused on the 20 outfield players from each match’s starting line-up who played the entire first half of each match. This approach was chosen because previous research has shown that player substitutions can significantly affect the tactical, physical, and technical performance of teams [32,33]. Given the high number of substitutions made by coaches during the second half, the study limited data analysis to the first half of each match to maintain consistency in the data collected and to minimize the impact of these changes on the analysis. Therefore, it was considered 35 min for U13 (an official match lasts for 70 min) and 40 min for U15 (an official match lasts for 80 min). The match sessions consisted of an 11 vs. 11 official match, on a 104 × 64 m pitch, with official rules. All players performed a 15 min standard warm-up consisting of ball possessing and dynamic stretching.

Before the beginning of each match, players were outfitted with a 10 Hz Catapult MinimaxX unit (MinimaxX S4, 10 Hz, Firmware 6.70, Catapult Innovations, Melbourne, Australia) which has been demonstrated to be valid and reliable [34]. The systems collected latitude and longitude coordinates, which were then extracted and resampled using an interpolation method to standardize the length of the time series. Subsequently, these coordinates were converted into metres using the Universal Transverse Mercator (UTM) coordinate system through specific coding routines [35]. The data were then smoothed with a 3 Hz Butterworth low-pass filter. To align the positional data with the field, a rotation matrix was applied, orienting the length of the playing field along the x-axis and the width along the y-axis. This matrix adjustment ensures that the players’ positional data are consistent with the spatial orientation of the playing field, as detailed in the methodology outlined by Pereira, Gonçalves [36].

### 2.3. Positioning Relations

**Figure 1:**
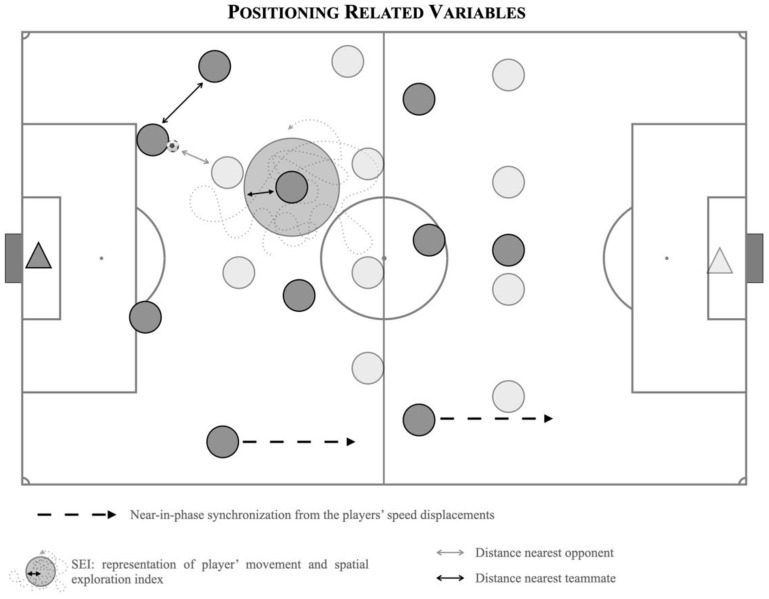
Representation of positional-related variables. Note: Dark grey circles represents one team, while light grey circles represents the other team.

The positional data of the players were used to determine the following variables (see Figure 1):

- Frequency of near-in-phase synchronization from the players’ speed displacements (expressed in % of time). Taking into consideration the all-possible intra-team dyads formed by the outfield teammates (45 dyads), the frequency of near-in-phase synchronization from the players’ speed displacements was processed (expressed in % of time) [10]. The Hilbert Transform [37] was used to compute the relative phase of the time series corresponding to the speed displacements of all dyads. Near-in-phase synchronization (i.e., % of time spent between −30° and 30° of relative phase) was used to access players’ interpersonal speed coordination.
- Spatial exploration index (SEI), which is processed by the calculation of the player’s mean position and then computing all distances from this average point to all datasets across the time series, and ending by computing the average value from all these distances [38].
- Distance to the nearest teammate and opponent expressed as absolute values (m), variability in these distances as expressed by the coefficient of variation (CV), and regularity in these distances expressed by the approximate entropy (ApEn) [39].

The ApEn has been used to assess the regularity in the players’ movement behaviour, and its values range from 0 to 2 (arbitrary units). From a processing approach, ApEn expresses the probability that the configuration of one segment of the data in a time series will allow the prediction of the configuration of another segment of the time series a certain distance apart. In practice, this technique may be used, for example, to identify if players’ positioning dynamics express a regular and predictable pattern which may, in turn, provide information regarding their tactical behaviour. The input values used to process the ApEn were 2 for the vector length (m) and 0.2 × SD for the tolerance (r) [40,41].

### 2.4. Statistical Analysis

To evaluate variations in positional performance during matches, each match half analysed in the study was divided into three equal segments, or thirds, and this division was utilized as a factor in the analysis. Descriptive data were presented as means ± standard deviation (SD). Before inferential statistics, the Shapiro–Wilk and Levene’s tests were performed to analyse whether the variables followed a normal distribution and verify the homogeneity of the variances, respectively. A two-way analysis of variance with repeated measures ANOVA [age group (U13 and U15) × half period (full, 1st, 2nd, and 3rd third)] was applied to test age and half period on the dependent variables. When significant main effects or interactions were achieved, Bonferroni post hoc analyses were performed to locate the pairwise. To estimate the strength of significant findings, effect sizes (ESs) were determined using Cohen’s *d_unbiased_* [42,43]. Effect size values were interpreted as follows: <0.20 represents a trivial effect, 0.20 to 0.49 is classified as a small effect, 0.50 to 0.79 corresponds to an intermediate effect, and 0.80 and higher is considered a large effect [44]. The analysis reports the effect size using eta squared (η^2^) for the main effects and interactions from the repeated measures ANOVA. For significant main effects or interactions, Cohen’s *d_unbiased_* was used to indicate the effect size for the pairwise post hoc comparisons. The statistical analyses were conducted using SPSS software v.26 for Windows (IBM Corp., Armonk, NY, USA), and the significance level was established at *p* ≤ 0.05.

## 3. Results

**Figure 2:**
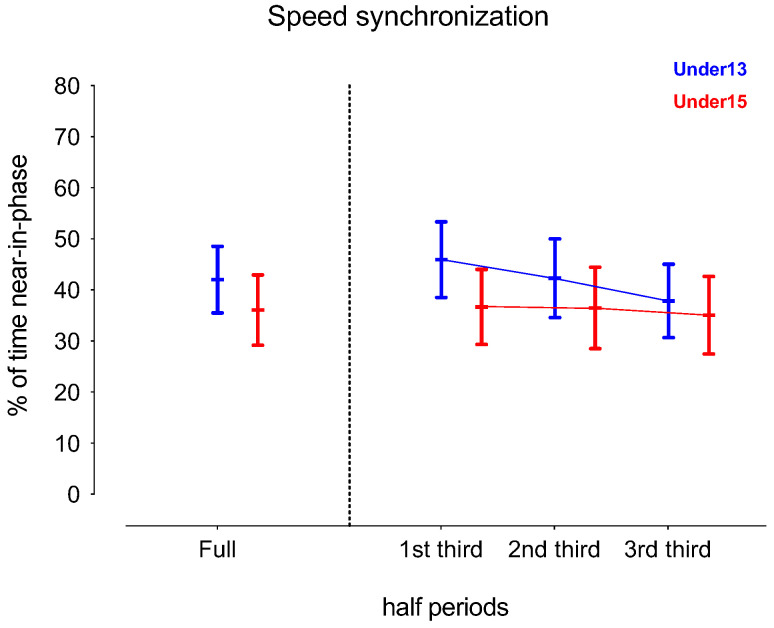
Descriptive values for players’ speed displacement synchronization according to the game periods (1st third, 2nd third, and 3rd third) and age groups (U13 and U15). Each dot represents an intra-team dyad value and the coloured error bars indicate mean ± standard deviation.

**Figure 3:**
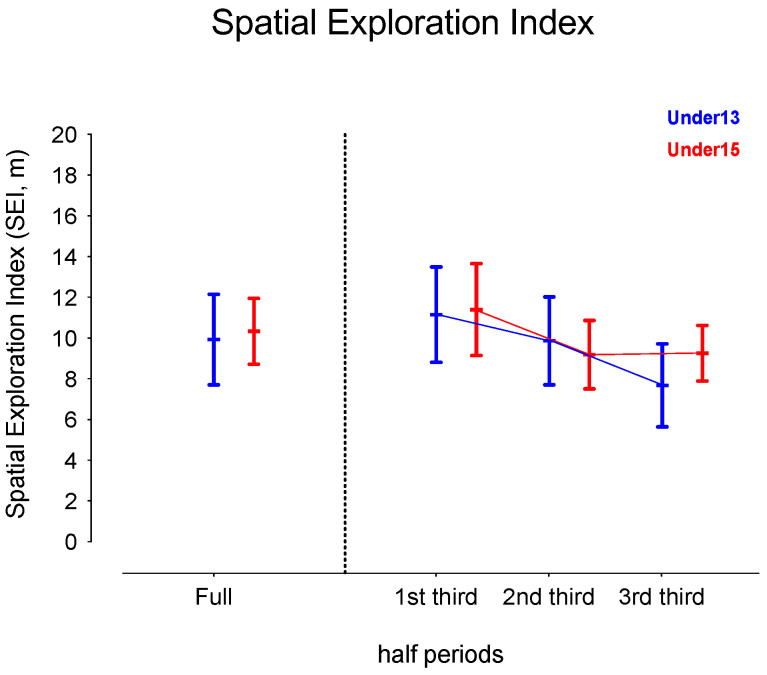
Descriptive values for the players’ spatial exploration index (SEI) according to the game periods (1st third, 2nd third, and 3rd third), age groups (U13 and U15), and their interactions. Each dot represents an intra-team dyad value and the coloured error bars indicate mean ± standard deviation.

**Figure 4:**
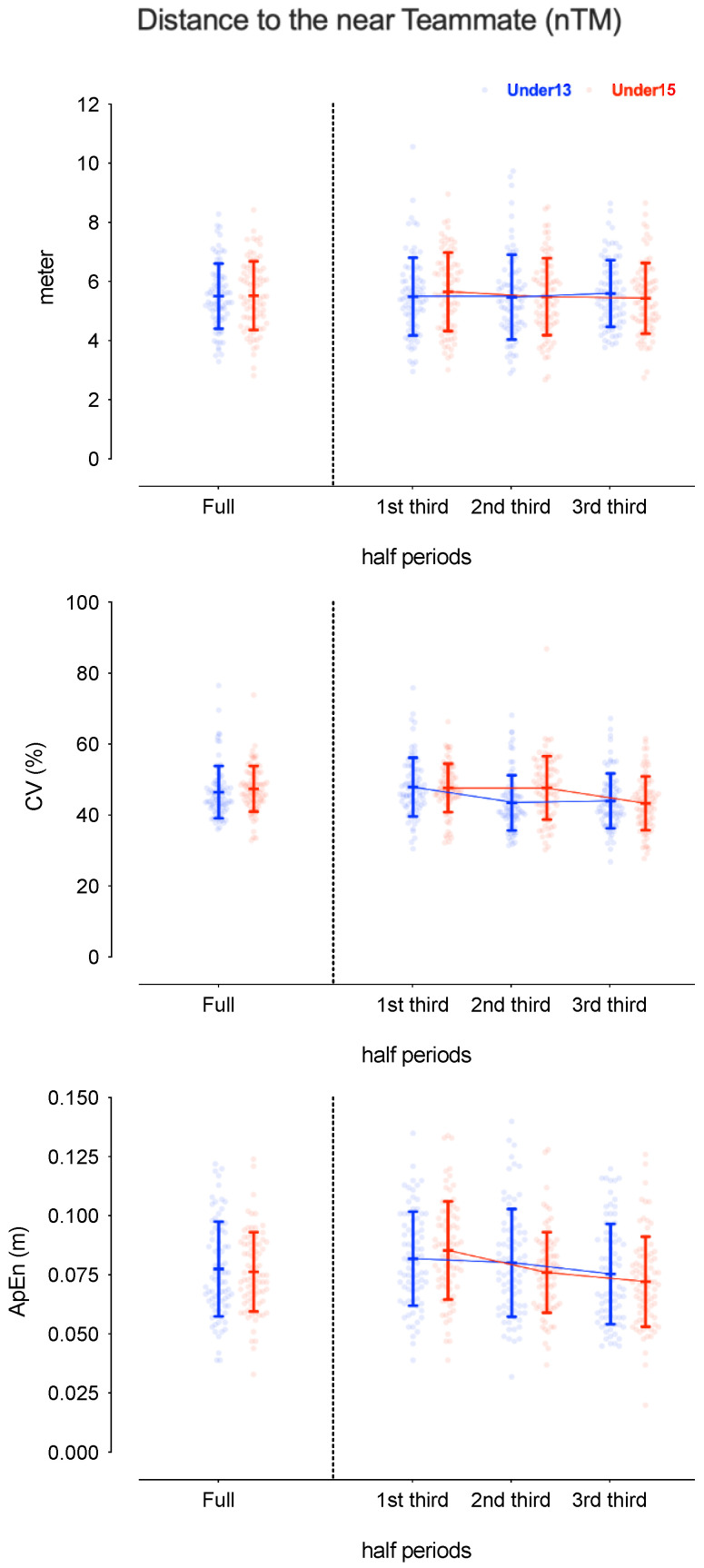
Descriptive values for players’ distance to the near teammate (nTM) according to the game periods (1st third, 2nd third, and 3rd third), age groups (U13 and U15), and their interactions. Each dot represents an intra-team dyad value and the coloured error bars indicate mean ± standard deviation. CV = coefficient of variation; ApEn = approximate entropy.

**Figure 5:**
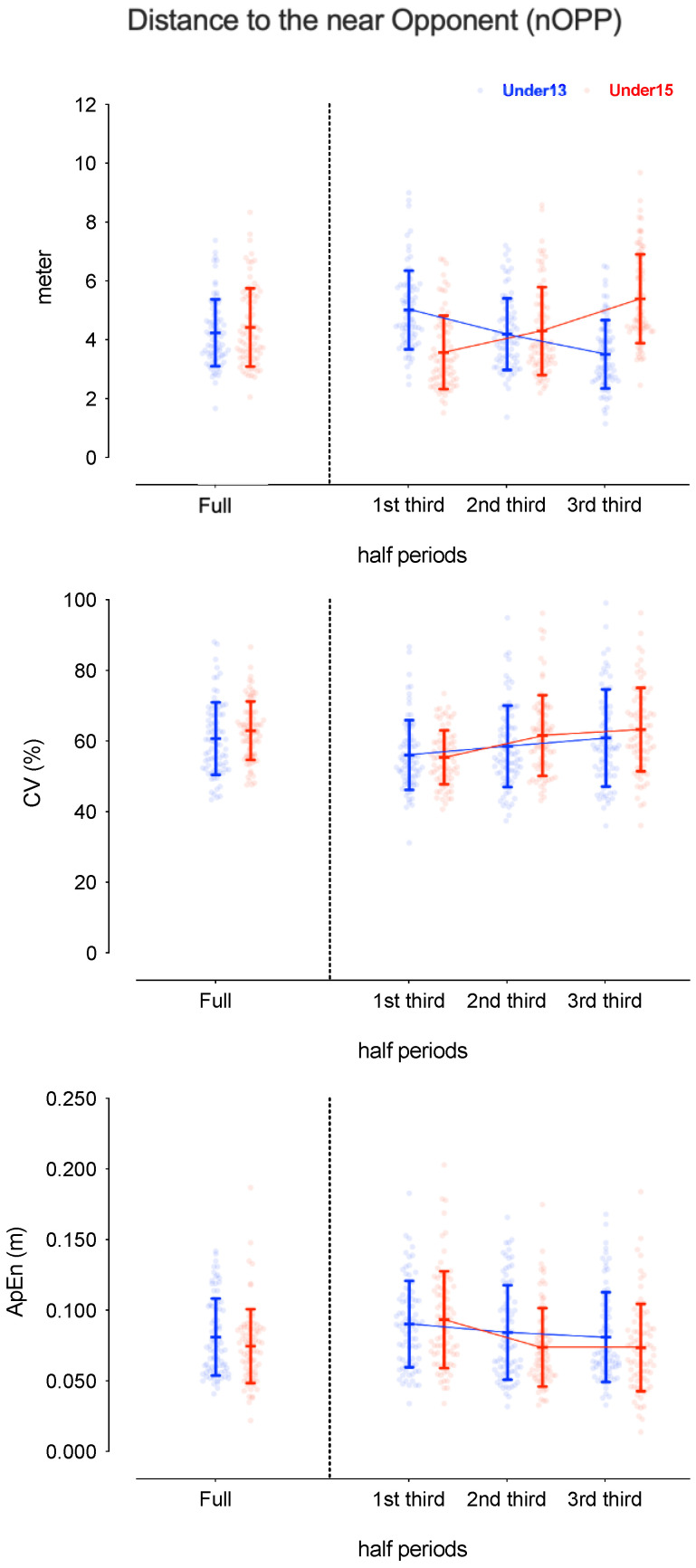
Descriptive values for players’ distance to the near opponent (nOPP) according to the game periods (1st third, 2nd third, and 3rd third), age groups (U13 and U15), and their interactions. Each dot represents an intra-team dyad value and the coloured error bars indicate mean ± standard deviation. CV = coefficient of variation; ApEn = approximate entropy.

**Figure 6:**
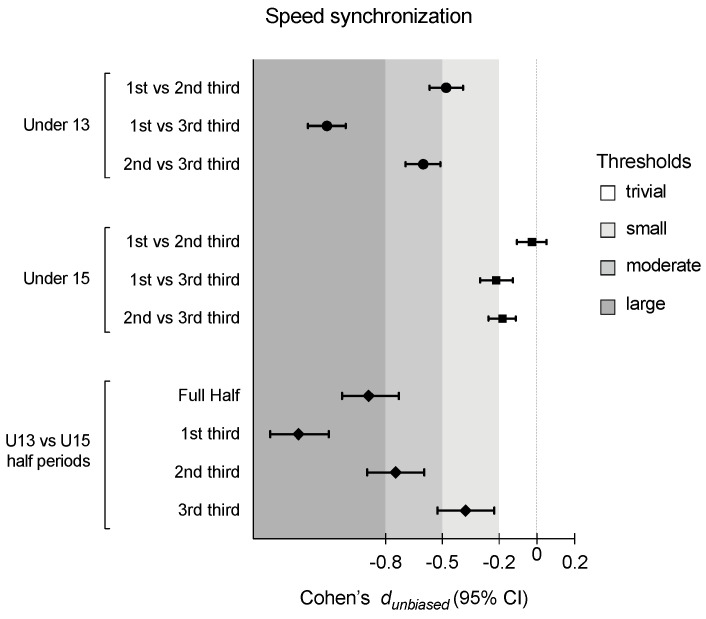
Cohen’s *d_unbiased_* differences for players’ speed displacement synchronization according to the game periods (1st third, 2nd third, and 3rd third), age groups (U13 and U15), and their interactions. Error bars indicate uncertainty in the true mean changes with 95% confidence intervals.

**Figure 7:**
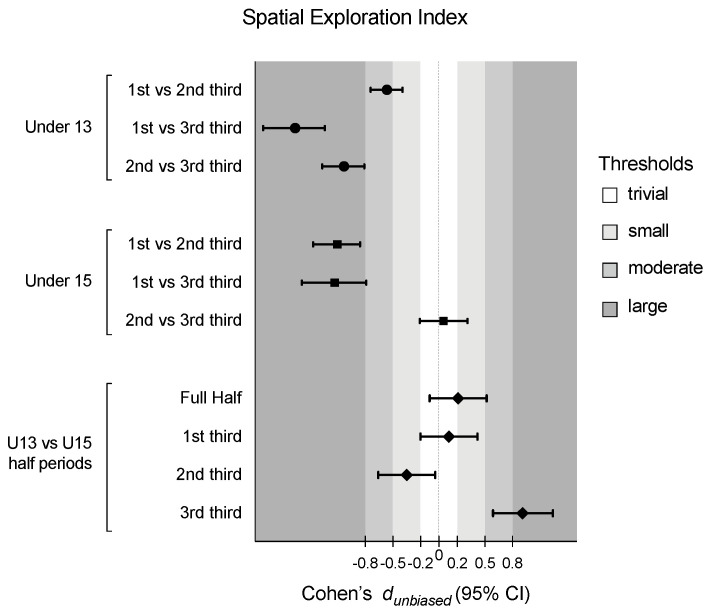
Cohen’s *d* differences for the players’ spatial exploration index according to the game periods (1st third, 2nd third, and 3rd third), age groups (U13 and U15), and their interactions. Error bars indicate uncertainty in the true mean changes with 95% confidence intervals.

**Figure 8:**
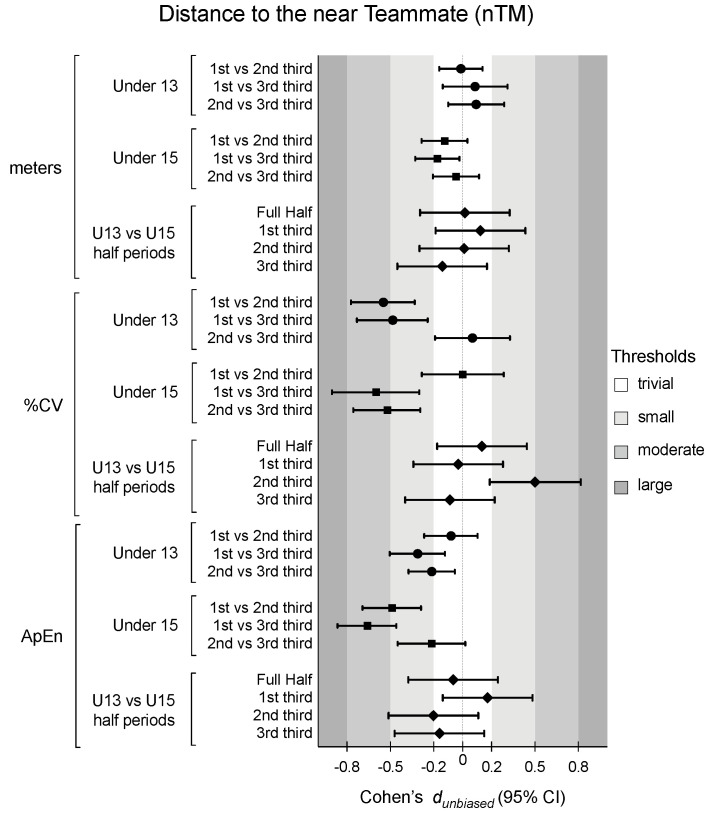
Cohen’s *d* differences for players’ distance to the near teammate (nTM) according to the game periods (1st third, 2nd third, and 3rd third), age groups (U13 and U15), and their interactions. Error bars indicate uncertainty in the true mean changes with 95% confidence intervals. CV = coefficient of variation; ApEn = approximate entropy.

**Figure 9:**
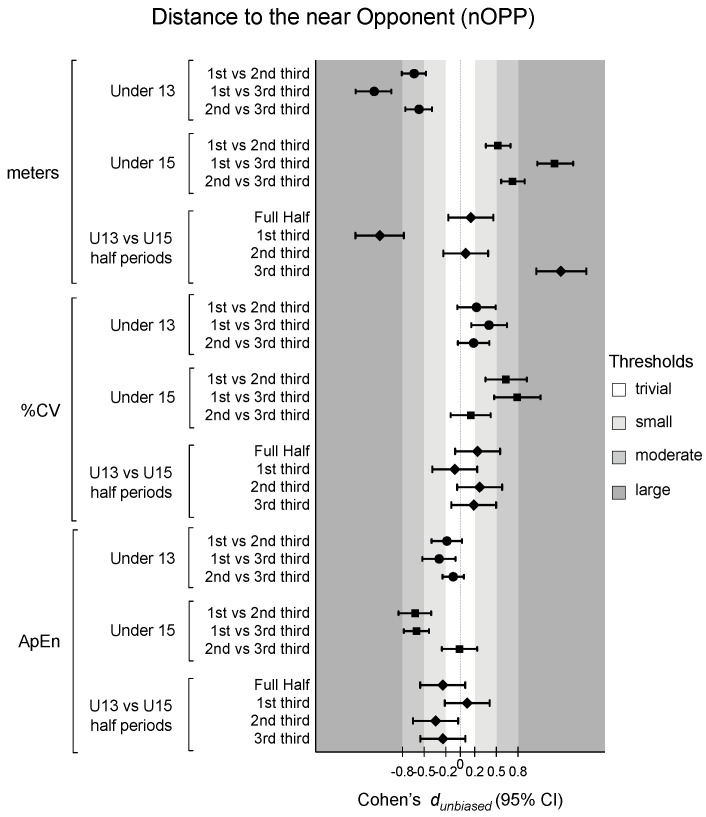
Cohen’s *d* differences for players’ distance to the near opponent (nOPP) according to the game periods (1st third, 2nd third, and 3rd third), age groups (U13 and U15), and their interactions. Error bars indicate uncertainty in the true mean changes with 95% confidence intervals. CV = coefficient of variation; ApEn = approximate entropy.

Table 1 presents the descriptive and inferential analysis for considered variables in both age groups and half periods. Figure 2, Figure 3, Figure 4 and Figure 5 depict the descriptive result for visual inspection analysis, and Figure 6, Figure 7, Figure 8 and Figure 9 depict the Cohen’s *d_unbiased_* result for respective pairwise comparison.

The analysis commenced by examining the interaction between the half period (with three levels: 1st third, 2nd third, and 3rd third) and age group (U13 vs. U15) on % of the time in near-in-phase speed synchronization. A two-way ANOVA with repeated measures revealed a significant interaction effect, with F = 109.5, *p* < 0.001, and η^2^_p_ = 0.13, indicating that the synchronization over time differed between the groups. The main effect of the half period was also significant, with F = 247.1, *p* < 0.001, and η^2^_p_ = 0.26, suggesting that the % of synchronization changed over time, regardless of the group assignment. Additionally, the main effect of the group was significant, with F = 142.3, *p* < 0.001, and η^2^_p_ = 0.17. This suggests that, overall, the U15 group exhibited a lower % of synchronization across all time points compared to the U13 group (see Table 1). Considering the post hoc analysis as well as Cohen’s *d_unbiased_* results, and comparing U13 vs. U15, U13 had significantly (*p* < 0.001) more % of synchronization in all considered half periods: a large effect for both the full half (Cohen *d_unbiased_* [95% CI]; −0.89 [−1.03; −0.73]) and the 1st third (−1.26 [−1.41; −1.10]); a moderate effect for the 2nd third (−0.75 [−0.91; −0.61]); and a small effect for the 3rd third (−0.38 [−0.53; −0.23]). The U13 period significantly decreased over the half while trivial to small results were identified for the U15 period in comparison (see Figure 2 and Figure 3).

The players’ SEI showed a significant effect on the period*age interaction, F = 27.3, *p* < 0.001 and η^2^_p_ = 0.15, and the half period, with F = 169.1, *p* < 0.001, and η^2^_p_ = 0.52 (see Table 1). U13 decreased their values over the match, from a small to large effect size, while U15 decreased only after the 1st third (1st vs. 2nd third: −1.10 [1.37; −0.87], and 1st vs. 3rd third: −1.13 [−1.49; −0.79], both large effect sizes). On the 3rd third, U15 showed higher values compared to U13 (see Figure 4 and Figure 5).

The players’ distance to the near teammate nTM was analysed from absolute values, metres, the coefficient of variation (%CV) as the magnitude of the variability, and approximate entropy (ApEn) as the magnitude of the structure variability. The absolute values were similar for both age groups and for all periods (see Table 1). However, the %CV showed significant differences in the period*age interaction, F = 7.4, *p* < 0.001 and η^2^_p_ = 0.05, and the half period, with F = 17.3, *p* < 0.001, and η^2^_p_ = 0.10 (see Table 1). The U13 group decreased from the 1st to 2nd third (1st vs. 2nd third: −0.55 [−0.77; −0.33]; and 1st vs. 3rd third: −0.48 [−0.73; −0.24]), while U15 decreased from the 2nd to 3rd third (1st vs. 3rd third: −0.60 [−0.90; −0.30]; and 2nd vs. 3rd third: −0.52 [−0.76; −0.29]). The ApEn also presented significant differences in the period*age interaction, F = 4.8, *p* = 0.01 and η^2^_p_ = 0.03, and the half period, F = 26.7, *p* < 0.001 and η^2^_p_ = 0.14 (see Table 1). Pairwise differences showed that U13 and U15 presented similar values. However, for U13, the 1st and 2nd third were similar, and the distance to nTM became more regular in the 3rd third (1st vs. 3rd third: −0.31 [−0.50; −0.12]; and 2nd vs. 3rd third: −0.21 [−0.37; −0.05]). U15 decreased the ApEn value across the match (see Figure 6 and Figure 7).

The players’ distance to the near opponent (nOPP), considering absolute values, revealed a significant interaction effect, with F = 293.3, *p* < 0.001, and η^2^_p_ = 0.65, indicating that the nOPP over time differed between the groups and match period, with F = 4.9, *p* = 0.01, and η^2^_p_ = 0.06, suggesting that nOPP changed over time (see Table 1). Additionally, while the main effect of the group was not significant, the pairwise differences presented a lower distance to nOPP for U15 in the 1st third (−1.11 [−1.45; −0.78]) and higher values in the 3rd third (1.39 [1.05; 1.74]). In fact, U13 decreased the distance to nOPP over the match while U15 increased during the same period (moderate to large effect size for both age groups). The %CV only revealed a significant half-period effect, with F = 19.7, *p* < 0.001, and η^2^_p_ = 0.11, where the values of nOPP increased over time for both groups (see Table 1). Finally, the ApEn presented significant differences in the period * age interaction, with F = 5.1, *p* = 0.01, and η^2^_p_ = 0.03, and the half period, with F = 29.9, *p* < 0.001, and η^2^_p_ = 0.14 (see Table 1). Pairwise differences showed that U13 and U15 presented similar values. However, for U15, the distance to nOPP becomes more regular right after the 1st third (1st vs. 2nd third: −0.62 [−0.85; −0.40]; and 1st vs. 3rd third: −0.60 [−0.77; −0.43]) (see Figure 7 and Figure 8).

## 4. Discussion

This study aimed to explore and compare changes in positioning performance among youth soccer players (U13 and U15) during an 11-a-side match. Generally, results from the U13 group indicated a decrease in synchronization speed and the amount of space explored, along with a reduction in distance to the nearest opponent (nOPP). For the U15 group, differences were primarily observed in the third period of the match, with a decrease in synchronization speed and in SEI, while the distance and regularity to the nOPP increased. When comparing both age groups, differences in synchronization speed were noted throughout the entire match, as well as in all periods, with higher values being observed in the U13 group. Additionally, differences between age groups became more pronounced as the game progressed, particularly in the second and third periods.

### 4.1. Analysis of U13 Positioning Variation across Time Periods

A major aim with younger age groups (i.e., from U5 to U14) is to develop players’ technical and coordination skills [45,46,47] while developing players’ understanding of the general (i.e., reject numerical inferiority, avoid numerical equality, and seek numerical superiority) and specific principles of play (i.e., offensive and defensive behaviours that guide individual, group, and collective movement behaviours) [48]. Developing such skills seems to be a determinant for future achievements in football competitive environments [49,50]. The development of such technical, coordinative, and tactical skills must be grounded in learning environments that foster decision-making skills, and competitive and cooperative interactions. In fact, decision-making and proper positioning seem to be related to talent in football [51]. Therefore, a meticulous and careful long-term plan is required to enhance the chances of youth players to progress in football. In line with this, a high number of football associations and researchers have been exploring which competitive formats may be more suitable for the different age groups [29,52]. For example, Sanchez, Ramirez-Campillo [53] compared U12 performance in 7-a-side, 8-a-side, and 11-a-side conditions and found higher external load in the larger format compared to the other two conditions. From the technical perspective, the seven-a-side format seems to elicit a greater number of actions when compared to the eight-a-side format in U12 [54]. A similar trend was found by Joo, Hwang-Bo [30] who explored the effects of using SSGs (8-a-side) in smaller (length × width, 68 × 47 m) or regular spaces (75 × 47 m) when compared to official matches (11-a-side, 75 × 47 m) in U12 Korean players. Altogether, the results of these studies seem to highlight that the 11-a-side format may be significantly complex for U12 players, who may not possess the technical (e.g., long pass ability) nor decision-making skills (i.e., the ability to scan the environment to perceive teammates’ and opponents’ positioning) that may allow them to successfully perform in such designs.

A similar conclusion may be drawn from the present study in the U13 age groups when analysing their tactical behaviour. That is, there was a decrease in synchronization speed, SEI, and distance to nOPP, while there was an increase in the regularity of the distance to the nearest opponent across the half thirds. These results suggest that as the match unfolds, there is a shift in the players’ focus from the collective movement behaviour towards the direct opponent. In fact, from the 1st third towards the 3rd third, there is a decrease of almost 1.5 m in the distance to the nOPP, which was followed by an increase in the regularity of this distance. In other words, players seem to become closer to their direct opponent, while maintaining this distance across the half-periods. Accordingly, younger age groups seem to be more focused on the ball and on the closest opponent than on the team’s collective approach [55], which may justify these results. Interestingly, these trends were more evident across thirds, suggesting that players were able to keep a collective strategy for the match during the first 15 min. Although anecdotally, as the coach’s instruction was not measured, the pre-match speeches are often focused on providing descriptions of players’ roles, emphasizing information about the opposition’s weakness, while providing information on how to collectively behave during the different game phases [23]. Thus, it may be plausible to assume that U13 players are able to follow a collective strategy within the first minutes of the match, after which it seems to fade into a more individual focus on the ball and the opponent. In fact, younger age groups attempt to solve the game problems by adopting an individual approach rather than a collective one [27].

From a practical point of view, governmental entities and national football associations must consider the type of competitive designs in youth age groups. For example, smaller formats may be more suitable for the U13 group. Alternatively, it may be important to add stoppage periods that may allow coaches to provide individual and collective feedback, allowing the players to adjust their tactical behaviours.

### 4.2. Analysis of U15 Positioning Variation across Time Periods

Older age group players seem to be more able to move and adjust to the competitive environment [56] by being able to identify the relevant information to unfold goal-directed behaviours as a result of better perceptual and cognitive skills [57]. In general, 11 vs. 11 formats are used from the U14 age groups above across different countries [29], which may suggest that this age is a point at which players might be able to perceive and act within complex competitive environments. The results from the present study seem to support this statement, as the U15 positional variables (e.g., SEI, distance to the nearest teammate, and ApEn in the distance to the nearest opponent) seem to be less affected across half-period thirds. For instance, most variations in players’ performance emerge in the 3rd third, with decreases in speed synchronization, SEI, and distance to the nTM, lower variability, and higher regularity in the distance to the NTM. In contrast, a bigger distance toward the nOPP was found. In general, these results point out that U15 can keep its performance constant for most variables across the first two thirds of the half. Based on this information, the transition to the 11 vs. 11 format may require a rest period around the middle of each half that may allow the players to reorganize their positioning. Still, a different strategy is depicted when compared to the U13 age group. That is, while in the U13 group, a decrease in the distance to the nOPP was found, an opposite trend was identified for the U15 group. Thus, it seems that with increased fatigue resulting from the competitive interactions, U15 adopts a more collective approach by decreasing the distance to the nTM and increasing it towards the nOPP. These findings are in line with the study of Coutinho, Gonçalves [58], who explored how U14 players’ positioning performance was affected during small-sided games by performing with additional muscular fatigue. The authors found a decrease in the distance between dyads, while also observing greater movement coordination. In addition, in this study, it was also found that there was a lower variation and higher regularity in the distance to the nTM for the U15 age group from the 1st to the 3rd third. A previous study showed higher values for the inter-team distance in the U15 age group than in the U13 group, which may reinforce these results. In contrast, a higher coefficient of variation in the nearest was found in both the 2nd and 3rd third when compared to the 1st third. This variability may act as a functional movement behaviour, because of the higher compactness (i.e., expressed by the lower distance to the nTM and SEI). In fact, variability in players’ movement behaviours has been considered fundamental to adjusting to the dynamic and unpredictable nature of competitive football settings [59].

### 4.3. Differences between U13 and U15

A wide body of research has been exploring differences between age groups from a tactical point of view. For example, Folgado, Lemmink [27] compared the performance of U9, U11, and U13 under three-a-side and four-a-side small-sided game formats. The three-a-side format revealed major differences in the distance between players, where the older players revealed a greater ability to use the pitch length, while similar distances between players were identified for the four-a-side format. Olthof, Frencken [28] compared U13, U15, U17, and U19 performances during five-a-side small-sided games while varying pitch dimensions (i.e., small, 40 × 30 m; and large, 68 × 47 m). The results showed a greater distance between players and the playing area in the U15 group when compared to the U13 group. The same trend was identified by a recent study comparing U13, U15, and U18 players’ positioning performance during a five-a-side small-sided game [31]. Older players revealed larger areas, and also a bigger distance between teams. The combined findings from these studies highlight that older players are more able to use the available space. In the present study, major differences between age groups were identified for speed synchronization and SEI. In this respect, higher values of synchronization speed were identified in all thirds for the U13 group. As previously noted, there is a higher trend towards following the ball movement in younger age groups [55], which may have contributed to such values. That is, this age group seems to be less prone to move collectively, but rather, they focus on the ball movement, and thus, it may be expected that both teams move as a result of the ball’s location. In contrast, the U15 group may possess higher tactical awareness that allows them to vary between moving collectively (e.g., staying compact while defending to press the opposition) or moving at different paces, rhythms, and directions (e.g., attempting to perform depth passes in the last third, whereas, one to two players may move close to the ball to drag defenders, with one or two sprinting to explore the space). Thus, the lower synchronization values in the U15 group may reflect this age group’s ability to understand each configuration of play. In fact, this group was less affected by the thirds. For example, the U13 group showed a clear trend towards decreasing the space explored as the thirds progressed, while the U15 group despite decreasing from the 1st third to the 2nd, was kept constant to the 3rd third. Younger age groups, such as the U13 group, are likely to adopt more individual strategies to solve game problems than explore collective movement solutions [27]. Consequently, and as the match unfolds, they may decrease the space exploration as a result of the lower collective commitment. In contrast, the U15 group revealed a decrease from the 1st to the 2nd third but kept the values constant to the 3rd third. The values from the 1st third may result from the inherent variability in team behaviours in the first 15 min, in which both teams may be exploring adaptive movement patterns [7]. However, as the match unfolds and the fatigue increases, U15 players may adopt more collective and stable behaviours [58].

## 5. Conclusions

Overall, it is important to be aware that exposing young players to 11 vs. 11 matches for long periods may not provide an appropriate learning environment, especially in the U13 age group. The high density of players and available space contributed to more variable and irregular behaviours across time, which can be depicted from the lower speed displacement % synchronization (i.e., collective variable) and higher SEI (i.e., individual variable). In contrast, the U15 group appears to be able to reveal positional adjustments over time, reflecting their higher tactical awareness. These findings highlight the necessity of tailoring youth football training and competition structures to suit the developmental needs and capabilities of various age groups, thereby optimizing learning and performance outcomes.

## Figures and Tables

**Table 1:** Descriptive and inferential analysis when comparing period effect (1st third × 2nd third; 1st third × 3rd third; and 2nd third × 3rd third), the age groups (U13 × U15), and also their interaction.

Variables	Age Group	Half Period				Repeated Measures Analysis								
						Period Effect (1st Third, 2nd Third, and 3rd Third)			Age Group Effect (U13 × U15)			Period * Age (Thirds × U13 and U15)		
		Full	1st Third	2nd Third	3rd Third	F	*p*	η^2^*_p_*	F	*p*	η^2^ *_p_*	F	*p*	η^2^ *_p_*
Speed synchronization % of time near-in-phase	U13	42.0 ± 6.5 ^#^	45.9 ± 7.4 ^a,b,#^	42.3 ± 7.7 ^c,#^	37.8 ± 7.2 ^#^	247.1	<0.001	0.26	142.3	<0.001	0.17	109.5	<0.001	0.13
	U15	36.1 ± 6.9	36.7 ± 7.4 ^b^	36.5 ± 8.0 ^c^	35.1 ± 7.6									
Spatial exploration index	U13	9.9 ± 2.2	11.1 ± 2.3 ^a,b^	9.9 ± 2.2 ^c, #^	7.7 ± 2.0 ^#^	169.1	<0.001	0.52	2.2	0.14	0.01	27.3	<0.001	0.15
	U15	10.3 ± 1.6	11.4 ± 2.3 ^a,b^	9.2 ± 1.7	9.3 ± 1.4									
Distance to the near teammate (nTM)														
Metres	U13	5.5 ± 1.1	5.5 ± 1.3	5.5 ± 1.4	5.6 ± 1.1	0.6	0.53	0.00	0.0	0.98	0.00	2.1	0.13	0.01
	U15	5.5 ± 1.2	5.7 ± 1.3 ^b^	5.5 ± 1.3	5.4 ± 1.2									
Coefficient of variation %	U13	46.5 ± 7.4	48.0 ± 8.3 ^a,b^	43.5 ± 7.8 ^#^	44.1 ± 7.7	17.3	<0.001	0.10	1.4	0.24	0.01	7.4	<0.001	0.05
	U15	47.5 ± 6.4	47.7 ± 6.8 ^b^	47.7 ± 8.9 ^c^	43.4 ± 7.6									
Approximate entropy	U13	0.078 ± 0.020	0.082 ± 0.020 ^b^	0.083 ± 0.023 ^c^	0.075 ± 0.021	26.7	<0.001	0.14	0.2	0.65	0.00	4.8	0.01	0.03
	U15	0.076 ± 0.017	0.085 ± 0.021 ^a,b^	0.076 ± 0.017 ^c^	0.072 ± 0.019									
Distance to the near Opponent (nOPP)														
Metres	U13	4.2 ± 1.1	5.0 ± 1.3 ^a,b,#^	4.2 ± 1.2 ^c^	3.5 ± 1.2 ^#^	4.9	0.01	0.03	0.9	0.35	0.01	293.3	<0.001	0.65
	U15	4.4 ± 1.3	3.6 ± 1.2 ^a,b^	4.3 ± 1.5 ^c^	5.4 ± 1.5									
Coefficient of variation %	U13	60.8 ± 10.3	56.1 ± 9.9 ^b^	58.6 ± 11.6	60.9 ± 13.8	19.7	<0.001	0.11	1.5	0.22	0.01	1.9	0.16	0.01
	U15	63.0 ± 8.3	55.5 ± 7.6 ^a,b^	61.6 ± 11.4	63.3 ± 11.8									
Approximate entropy	U13	0.081 ± 0.027	0.090 ± 0.031 ^b^	0.084 ± 0.033 ^#^	0.081 ± 0.032	24.9	<0.001	0.14	1.4	0.25	0.01	5.1	0.01	0.03
	U15	0.075 ± 0.026	0.094 ± 0.034 ^a,b^	0.074 ± 0.028	0.074 ± 0.031									

Post hoc analysis—period * age. ^#^ U13 full vs. U15 full; U13 1st third vs. U15 1st third; U13 2nd third vs. U15 2nd third; U13 3rd third vs. U15 3rd third. ^a^ 1st third vs. 2nd third. ^b^ 1st third vs. 3rd third. ^c^ 2nd third vs. 3rd third.

## Data Availability

In order to protect the subjects’ confidentiality and privacy, data are only available upon request. Interested researchers may contact the corresponding author.
